# PPARG2 Pro12Ala and TNF*α* -308G>A Polymorphisms Are Not Associated with Heart Failure Development in Patients with Ischemic Heart Disease after Coronary Artery Bypass Grafting

**DOI:** 10.1155/2019/1932036

**Published:** 2019-06-02

**Authors:** Izabela Wojtkowska, Tomasz A. Bonda, Andrzej Tysarowski, Katarzyna Seliga, Janusz A. Siedlecki, Maria M. Winnicka, Janina Stępińska

**Affiliations:** ^1^Institute of Cardiology, Intensive Cardiac Therapy Clinic, Alpejska St., 04-628 Warsaw, Poland; ^2^Medical University of Bialystok, Department of General and Experimental Pathology, Mickiewicza 2c, 15-222 Bialystok, Poland; ^3^Institute of Oncology, Department of Molecular and Translational Oncology, Wawelska 15B St., 02-034 Warsaw, Poland

## Abstract

TNF*α* and PPAR*γ* are important modulators of metabolism, inflammation, and atherosclerosis. Coronary artery disease is the leading cause of heart failure (HF). The aim of the study was to assess whether polymorphisms of the* TNFα* (-308G>A) and* PPARG2* (Pro12Ala) genes are associated with the risk of developing HF by patients with ischemic heart disease.* Methods*. 122 patients without HF (aged 63 ± 8.8 years, 85% males) with confirmed coronary artery disease qualified for coronary bypass grafting were enrolled in the study. After the procedure, they were screened for cardiac parameters. Those with elevated NT-proBNP or diminished left ventricular ejection fraction during follow-up were assigned to the HF group (n=78), and the remaining ones to the non-HF group (n=44). The* TNFα* -308G>A and* PPARG*2 Pro12Ala polymorphisms were detected using the TaqMan method.* Results*. The distributions of* TNFα* -308G>A and* PPARG*2 Pro12Ala did not differ between the HF and non-HF groups (-308G>A: 16% vs. 11.4% of alleles; Pro12Ala: 23.9% vs. 20.5% of alleles, respectively). IL-6 concentration in the plasma of* TNFα* A-allele carriers at months 1 and 12 after CABG was higher in the HF group compared to the non-HF group (1 month after CABG: 5.3 ± 3.4 vs. 3.1 ± 2.9, p<0.05; 12 months after CABG: 4.2 ± 3,9 vs. 1.4 ± 1.2, p<0.01, respectively). Both polymorphisms were not related to changes in the plasma TNF*α* concentration or other parameters related to HF.* Conclusions*. Our study did not reveal any correlation between the* PPARG2* Pro12Ala and* TNFα* -308G>A polymorphisms and development of HF in patients with ischemic heart disease after coronary bypass grafting.

## 1. Introduction

Coronary heart disease (CHD) plays a critical role in the development of heart failure (HF). Nearly two-thirds of HF cases are attributed to underlying coronary artery atherosclerosis. An important aim of the CHD treatment is to prevent HF-associated morbidity and mortality. Progression of atherosclerosis is the main reason for development of CHD and is influenced by numerous factors such as high plasma LDL cholesterol concentration, blood glucose level, inflammation, and oxidative stress [1]. Many of the above factors have proven to be closely related to the polymorphisms of the peroxisome proliferator-activated receptor (*PPAR*) gene [2]. In recent years, there has been a growing interest in the link between* PPAR *gene polymorphisms, including* PPARA *intron 7G/C,* PPARD *+294T/C,* PPARG2 *Pro12Ala and C161T, and CHD risk [3–8], but data from these single studies have not provided consistent results. The data may not have sufficient statistical power to reveal relatively weak dependencies or allow analyses in specific populations. Moreover, meta-analyses including large populations have not provided any clear evidence for association between* PPAR *polymorphisms and coronary artery disease [9–11]. The most frequent* PPARG2 *polymorphism in Caucasian population (about 25%) is the Pro12Ala polymorphism in which cytosine is exchanged for guanine in codon 12, resulting in proline substitution by alanine in the PPAR*γ* protein [2]. This change has been reported to reduce the transcription of target genes, including genes regulating inflammation, and may be related to reduced synthesis of tumor necrosis factor *α* (TNF*α*) [2, 12, 13]. The* PPARG2 *gene is expressed mainly in the adipose tissue and thus may influence fatty acid turnover and cytokine release from adipocytes.* PPARG2 *expression has also been found in atherosclerotic lesions and macrophages, suggesting that* PPARG2 *may influence atherogenic processes [14, 15]. TNF*α* and PPAR*γ* are able to influence the myocardium through regulation of inflammatory cytokine release and metabolic modulation. However, their effect on the progression of HF is unclear.

The aim of the presented study was to determine a link between the polymorphisms (-308G>A (rs1800629) in* TNFα *and Pro12Ala (rs1801282) in* PPARG2*) and development of HF in patients with ischemic heart disease subjected to coronary artery bypass surgery (CABG).

## 2. Methods

Patients qualified for surgical treatment of coronary artery disease and without HF manifestations were enrolled in the study. All patients underwent coronary angiography confirming significant atherosclerotic lesions in the coronary arteries. Fifty-one patients (42%) had a history of myocardial infarction. Only patients with normal left ventricular ejection fraction (LVEF) in echocardiography and normal NT-proBNP were included, while patients with diabetes mellitus and valvular heart disease were excluded from the study. The protocol for patient qualification and follow-up was the same as described in our previous papers [16, 17].

The clinical status, biochemical tests, resting transthoracic echocardiography, and 6-minute walk test (6MWT) were performed before CABG and at 3 time points during the follow-up: 1, 12, and 24 months after the procedure. Patients who had elevated NT-proBNP (>400 pg/ml) or decreased LVEF (< 40%) during follow-up visits were assigned to the HF group, while patients without NT-proBNP elevation or LVEF decrease were assigned to the control (non-HF) group.

Blood samples were drawn at baseline and during each follow-up evaluation. Serum concentrations of IL-6 and TNF*α* were measured using solid-phase sandwich enzyme-linked immunosorbent assay kits (HS600B, R&D Systems) according to the manufacturer's guidelines.

Genomic DNA was isolated from blood leukocytes. Genotyping evaluating the* TNFα*-308G>A (rs1800629) and* PPARG2 *Pro12Ala (rs1801282) polymorphisms was performed with TaqMan probes using the Abi Prism 7500 Fast apparatus (Applied Biosystems).

### 2.1. Ethics Statement

The procedures followed in the study were conducted ethically according to the principles of the World Medical Association Declaration of Helsinki and Ethical Standards in Sport and Exercise Science Research. All procedures were approved by the Ethics Committee of the Regional Medical Chamber in Warsaw [IK NP-0021/13/998/2007]. Informed consent was obtained from all participants.

### 2.2. Statistics

Data was presented as mean ± SD for quantitative variables or percent of study group for qualitative variables. Specific parameters of both groups and changes in parameter values during follow-up were compared using chi-square test and one- and two-way ANOVA with post hoc tests. Chi-square test was used for evaluation of allele distribution in both groups. A value of p < 0.05 was considered statistically significant. Analysis was performed using Statistica 12 (StatSoft, Inc. 2014).

## 3. Results

A total of 122 Caucasian patients (aged 63 ± 8.8 years, 85% males) were qualified to the study. During post-CABG follow-up, the criteria for diagnosing HF were fulfilled in 78 patients (HF group), while the remaining 44 were assigned to the non-HF group. Demographic and initial clinical characteristics of the study groups are presented in Table 1.

**Figure 1:**
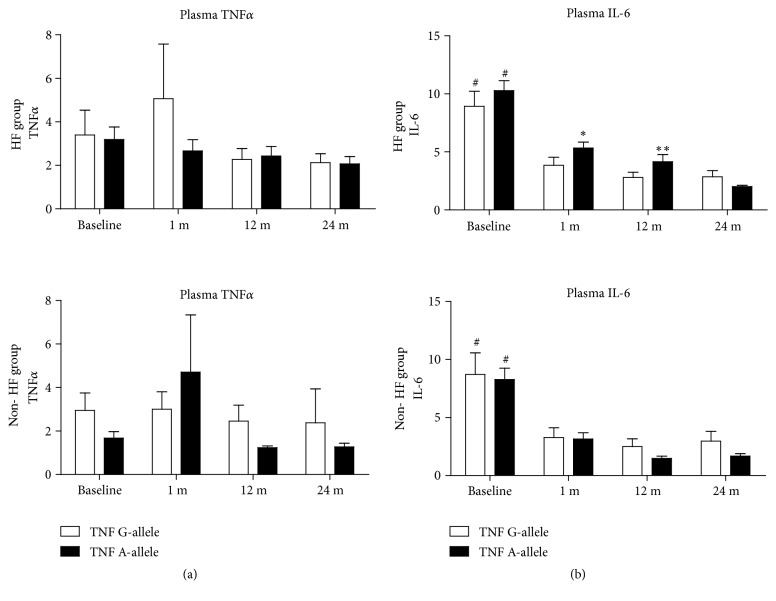
Plasma TNF*α* (panel (a)) and IL-6 (panel (b)) concentrations in the HF (upper graphs) and non-HF (lower graphs) groups in relation to the presence of specific alleles of* TNF* (position -308): G is the dominating isoform. There were only 3 AA homozygotes; thus AA were grouped together with GA into one “A-allele” subgroup, while the “G-allele” subgroup contained GG homozygotes only. Plasma TNF*α* concentrations were not statistically different between the HF and non-HF groups and were not affected by the* TNF*-308 polymorphism. IL-6 was significantly higher at baseline in both groups (# p<0.001 vs. follow-up results in the same subgroup). IL-6 levels were higher in the HF carriers of A-allele at 1-month and 12-month follow-up visits compared to the non-HF carriers of A-allele (panel (b) black bars; *∗* p<0.05, *∗∗*p<0.01). Bars represent mean, and whiskers represent SEM.

**Figure 2:**
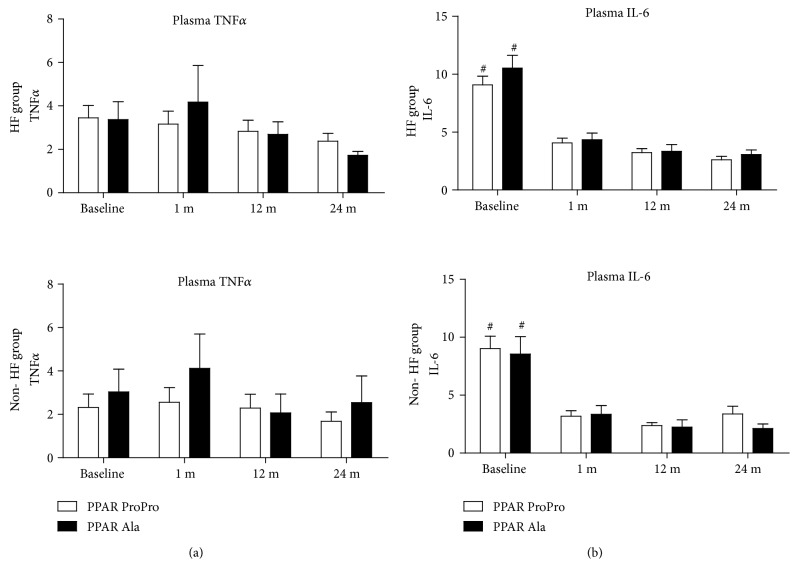
Plasma TNF*α* (panel (a)) and IL-6 (panel (b)) concentrations in the HF (upper graphs) and non-HF (lower graphs) groups in relation to the presence of specific alleles of* PPAR* Pro12Ala. The ProPro subgroup contains homozygotes with dominating isoform, while the Ala subgroup includes carriers of the mutated Ala allele: AlaAla homozygotes and ProAla heterozygotes. There were no differences in the cytokine profiles between the C-allele (Ala) and G-allele (Pro) subgroups in either the HF or the non-HF group. Baseline IL-6 levels were higher than follow-up measurements in all subgroups (# p<0.05 vs. remaining values in the same group and genotype). Bars represent mean, and whiskers represent SEM.

The distribution of the SNP alleles of* TNFα *and* PPARG2 *was comparable in the HF and non-HF groups (Table 2). The plasma levels of TNF*α* and IL-6 were not related to the* PPARG2 *or* TNF *polymorphisms before CABG and during follow-up. The profiles of plasma levels of TNF*α* and IL-6 were not significantly different between the HF and non-HF groups at each individual follow-up step. In both groups, no influence of the* TNFα *or* PPARG2 *genotype on the levels of TNF*α* was found (Figure 1). IL-6 concentration in the plasma of* TNFα *A-allele carriers at months 1 and 12 after CABG was higher in the HF group compared to the non-HF group (1 month after CABG: 5.3 ± 3.4 vs. 3.1 ± 2.9, p<0.05; 12 months after CABG: 4.2 ± 3,9 vs. 1.4 ± 1.2, p<0.01, respectively) (Figure 2). Plasma IL-6 concentration was higher before CABG, subsequently decreased one month after the procedure, and remained at a stable level in both the HF and non-HF groups irrespective of the* TNFα *and* PPARG2 *genotype (p<0.05; Figure 2).

In both groups, the* TNFα *polymorphism was not significantly correlated with any clinical or other biochemical parameters.* PPARG2 *Ala was related to a longer distance in the 6-minute walk test, but only in patients of the HF group before and 1 month after CABG (424 ± 62 m in* PPARG2* Ala vs. 394 ± 59 m in* PPARG2 *ProPro before CABG, p=0.025, and 417 ± 100 m vs. 383.4 ± 84 m, p=0.01, respectively). Other clinical and laboratory parameters were not related to the* PPARG2 *allele.

## 4. Discussion

Our results suggest a lack of significant relationship between the* TNFα*-308G>A and* PPARG2 *Pro12Ala polymorphisms and development of HF after CABG. The* TNFα *A-allele has been shown to promote expression of TNF*α*; however, in human studies, it has not been associated with significantly higher concentrations of TNF*α* in blood [18–21]. The presence of the A-allele has been correlated with inflammatory processes, higher C-reactive protein (CRP) levels, and development of metabolic syndrome, but in clinical observations this effect is very often lost due to multiple confounding factors [21–23]. As shown by a recent meta-analysis, A-allele carriers are more susceptible to developing ischemic heart disease [24]; however, there are also reports describing a protective role of this polymorphism. We observed a similar distribution of both G and A alleles of* TNFα* in the HF and non-HF groups. CRP levels did not differ between the carriers of the above alleles, and plasma TNF*α* levels were similar between the AA+AG and GG carriers. We found that patients with HF carrying the A-allele had significantly higher plasma IL-6 concentrations than A-allele carriers of the non-HF group. This may reflect a link between the proinflammatory IL-6 and this genetic variant of* TNFα* in patients with reduced cardiac function, even though this effect seems to be insignificant from the clinical point of view.

Association between the* PPARG2 *Pro12Ala polymorphism and development of HF has not been described previously, but has been investigated for its association with the development of atherosclerosis, including coronary atherosclerosis. In the Caucasian population of A homozygotes, a significantly increased risk of coronary artery disease has been reported [10].

The single nucleotide polymorphism (C>G) in codon 12 of exon B of the* PPARG2 *gene results in proline substitution by alanine in the PPAR*γ* protein, which is responsible for a reduced binding activity of PPAR*γ* to the cognate promoter element and a reduced ability to transactivate responsive promoters [25]. The presence of the Ala allele has been linked to increased sensitivity to insulin and reduced risk of developing type 2 diabetes mellitus, most probably resulting from changes in the adipocyte function, which is characterized by suppression of lipolysis and release of free fatty acids, as well as secondarily improved glucose utilization by muscles [2, 26].* PPARG2 *is expressed in the adipose tissue; thus the hypothetical consequences of the Pro12Ala polymorphism for the myocardium seem to be indirect, related to changes in lipid turnover and adipokine release [27], or attributed to the development of coronary atherosclerosis. Our study was conducted in a population with significant stenoses in the epicardial coronary arteries, and none of the patients had reduced systolic function of the left ventricle before the procedure. Moreover, the BMI of both study groups was at the same level; thus we presumed that the* PPARG2 *polymorphism should exert its effect not through an ischemic mechanism, but through endo/paracrine signaling engaging adipokines. We examined the profiles of TNF*α* and IL-6—the two adipokines involved in the pathogenesis of HF—but we did not find any difference in the TNF*α* and IL-6 levels between the dominant* PPARG2 *ProPro genotype and Ala carriers before CABG or during follow-up. In patients with HF, the Ala allele was associated with a longer distance in the 6-minute walk test. Previous reports have linked the Ala allele with the development of more effective short-term anaerobic muscle performance and strength [28], but since no difference between the genotypes was present in the non-HF group, it should not be explained by the* PPARG2 *Pro12Ala polymorphism.

We did not find correlations between the* PPARG2 *Pro12Ala and* TNFα*-308G>A polymorphisms and development of HF in patients with ischemic heart disease after coronary bypass grafting.

## Figures and Tables

**Table 1:** Initial characteristics of the investigated groups.

	HF	Non-HF	P
	(n=78)	(n=44)	
Age (y)	65.1 ± 8.4	59.6 ± 8.7	NS
BMI (kg/m^2^)	27.0 ± 3.5	27.9 ± 3.4	NS
Smokers	16 (20%)	4 (9%)	NS
Hypertension	48 (62%)	28 (63%)	NS
History of MI	35 (45%)	15 (35%)	NS
Systolic BP (mmHg)	132.2 ± 12.3	127.7 ± 9.0	0.035
Diastolic BP (mmHg)	79.5 ± 5.0	79.2 ± 4.70	NS
LVEDV (cm)	5.2 ± 0.3	5.1 ± 0.8	NS
LV EF (%)	59 ± 4.5	59 ± 3.5	NS
6-MWT (m)	405 ± 62	446 ± 63	0.0007
Blood count:			
RBC	4.3 ± 0.7	4.58 ± 0.6	0.031
HGB	13.3 ± 2.0	14 ± 1.9	NS
HCT	38.6 ± 5.8	40.32 ± 5.3	NS
WBC	7.9 ± 3.0	7.42 ± 2.5	NS
PLT	192.8 ± 59.9	186.64 ± 46.5	NS
Electrolytes:			
Na (mmol/L)	139.8 ± 14.7	141.8 ± 2.8	NS
K (mmol/L)	4.5 ± 0.4	4.5 ± 0.4	NS
CRP (mg/L)	7.1 ± 8.1	6.6 ± 9.0	NS
TNF (pg/mL)	3.3 ± 3.9	2.6 ± 3.8	NS
IL-6 (pg/mL)	9.4 ± 5.4	8.6 ± 6.0	NS
NT-proBNP (pg/mL)	212.4 ± 101	198.3 ± 111	NS
eGFR (mL/min/1.73m^2^)	74 ± 13.1	77.4 ± 11.0	NS
Total cholesterol (mmol/L)	4.1 ± 0.9	4.0 ± 0.8	NS
LDL-cholesterol (mmol/L)	2.2 ± 0.8	2.2 ± 0.7	NS
HDL-cholesterol (mmol/L)	1.4 ± 0.3	1.2 ± 0.3	0.016
Triglycerides (mmol/L)	1.3 ± 0.6	1.4 ± 0.6	NS
Medications:			
Aspirin	100%	100%	NS
Beta-blockers	100%	100%	NS
ACE-I	91%	93%	NS
ARB	7 %	0 %	NS
Aldosterone antagonists	11 %	0 %	0.007
Diuretics	19 %	9 %	NS
Statins	99 %	98%	NS

**Table 2:** Allele distribution in the heart failure (HF) and non-heart failure (Non-HF) groups.  *∗*  *χ*^2^ test.

		HF (78)	Non-HF (44)	P value*∗*
PPARG2 Pro12Ala	CC (ProPro)	48 (61.5%)	25 (57%)	
	CG (ProAla)	28 (35.9%)	17 (38.9%)	
	GG (AlaAla)	2 (2.6%)	2 (4.6%)	
	C allele %	79.5	76.1	NS
	G allele %	20.5	23.9	

TNF -308G/A	GG	56 (71.8%)	34 (77.3%)	
	GA	19 (24.4%)	10 (22.7%)	
	AA	3 (3.8%)	0	
	G allele %	84	88.6	NS
	A allele %	16	11.4	

## Data Availability

The source data (table containing the clinical parameters, concentrations of TNF*α* and IL-6, and results of the polymorphisms) are available from the corresponding author upon request.

## Additional Points

*Study Limitations*. A significant number of patients was lost during follow-up: 8 (6.5%) patients 12 months after CABG and 40 (32%) after two years.
